# Lewy body disease or diseases with Lewy bodies?

**DOI:** 10.1038/s41531-021-00273-9

**Published:** 2022-01-10

**Authors:** Kateřina Menšíková, Radoslav Matěj, Carlo Colosimo, Raymond Rosales, Lucie Tučková, Jiří Ehrmann, Dominik Hraboš, Kristýna Kolaříková, Radek Vodička, Radek Vrtěl, Martin Procházka, Martin Nevrlý, Michaela Kaiserová, Sandra Kurčová, Pavel Otruba, Petr Kaňovský

**Affiliations:** 1 Department of Neurology, University Hospital, Palacky University, Olomouc, Czech Republic; 2 Department of Neurology, Faculty of Medicine and Dentistry, Palacky University, Olomouc, Czech Republic; 3 Department of Pathology and Molecular Medicine, 3rd Faculty of Medicine, Charles University, Prague, Czech Republic; 4 Department of Neurology, Santa Maria University Hospital, Terni, Italy; 5 The Neuroscience Institute, Department of Neurology and Psychiatry, University of Santo Tomás Hospital, Manila, Philippines; 6 Department of Clinical and Molecular Pathology, Faculty of Medicine and Dentistry, Palacky University, Olomouc, Czech Republic; 7 Department of Clinical Genetics, University Hospital, Palacky University, Olomouc, Czech Republic

**Keywords:** Neurodegeneration, Parkinson's disease

## Abstract

The current nosological concept of α-synucleinopathies characterized by the presence of Lewy bodies (LBs) includes Parkinson’s disease (PD), Parkinson’s disease dementia (PDD), and dementia with Lewy bodies (DLB), for which the term “Lewy body disease” (LBD) has recently been proposed due to their considerable clinical and pathological overlap. However, even this term does not seem to describe the true nature of this group of diseases. The subsequent discoveries of α-synuclein (αSyn), *SNCA* gene, and the introduction of new immunohistochemical methods have started intensive research into the molecular-biological aspects of these diseases. In light of today’s knowledge, the role of LBs in the pathogenesis and classification of these nosological entities remains somewhat uncertain. An increasingly more important role is attributed to other factors as the presence of various LBs precursors, post-translational αSyn modifications, various αSyn strains, the deposition of other pathological proteins (particularly β-amyloid), and the discovery of selective vulnerability of specific cells due to anatomical configuration or synaptic dysfunction. Resulting genetic inputs can undoubtedly be considered as the main essence of these factors. Molecular–genetic data indicate that not only in PD but also in DLB, a unique genetic architecture can be ascertained, predisposing to the development of specific disease phenotypes. The presence of LBs thus remains only a kind of link between these disorders, and the term “diseases with Lewy bodies” therefore results somewhat more accurate.

## Introduction

The current approach to the classification of neurodegenerative parkinsonism is based principally on the results of the neuropathological examination. Nosology includes Parkinson’s disease (PD), Parkinson’s disease dementia (PDD), dementia with Lewy bodies (DLB), multiple system atrophy (MSA), progressive supranuclear palsy (PSP), corticobasal degeneration (CBD), frontotemporal lobar degeneration (FTLD), and Alzheimer’s disease (AD). All these disorders are clinically characterized by the validated clinical diagnostic criteria. Practically all clinical criteria differentiate “probable”, “possible”, and “definite” degrees of diagnostic certainty.

A more modern classification based on molecular pathology includes categories such as alpha-synucleinopathies, either neuronal (PD, PDD, DLB) or oligodendroglial (MSA), tauopathies (also called FTLD-tau; PSP, CBD, argyrophilic grain disease, AGD, primary age-related tauopathy, PART, and globular glial tauopathy, GGT), FTLD with inclusions of proteins TDP-43 (FTLD-TDP), FUS (FTLD-FUS), or other with immunoreactivity for components of the ubiquitin-proteasome system (FTLD-UPS), and AD.

**Fig. 1:**
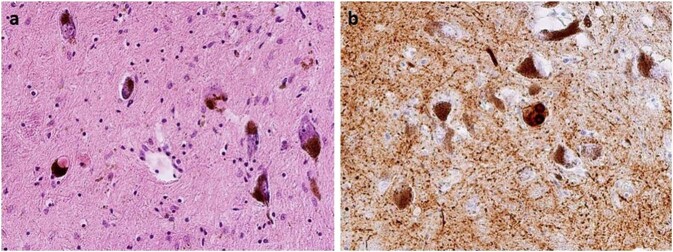
Pure alpha-synucleinopathy in typical Parkinson’s disease phenotype. **a** Classical Lewy bodies in the pigmented neurons of substantia nigra, HE staining, original magnification ×200. **b** Pathological deposits of α-synuclein in substantia nigra—Lewy bodies, granular cytoplasmic positivity, and dystrophic neurites, stained with a monoclonal antibody against α-synuclein, original magnification ×200.

**Fig. 2:**
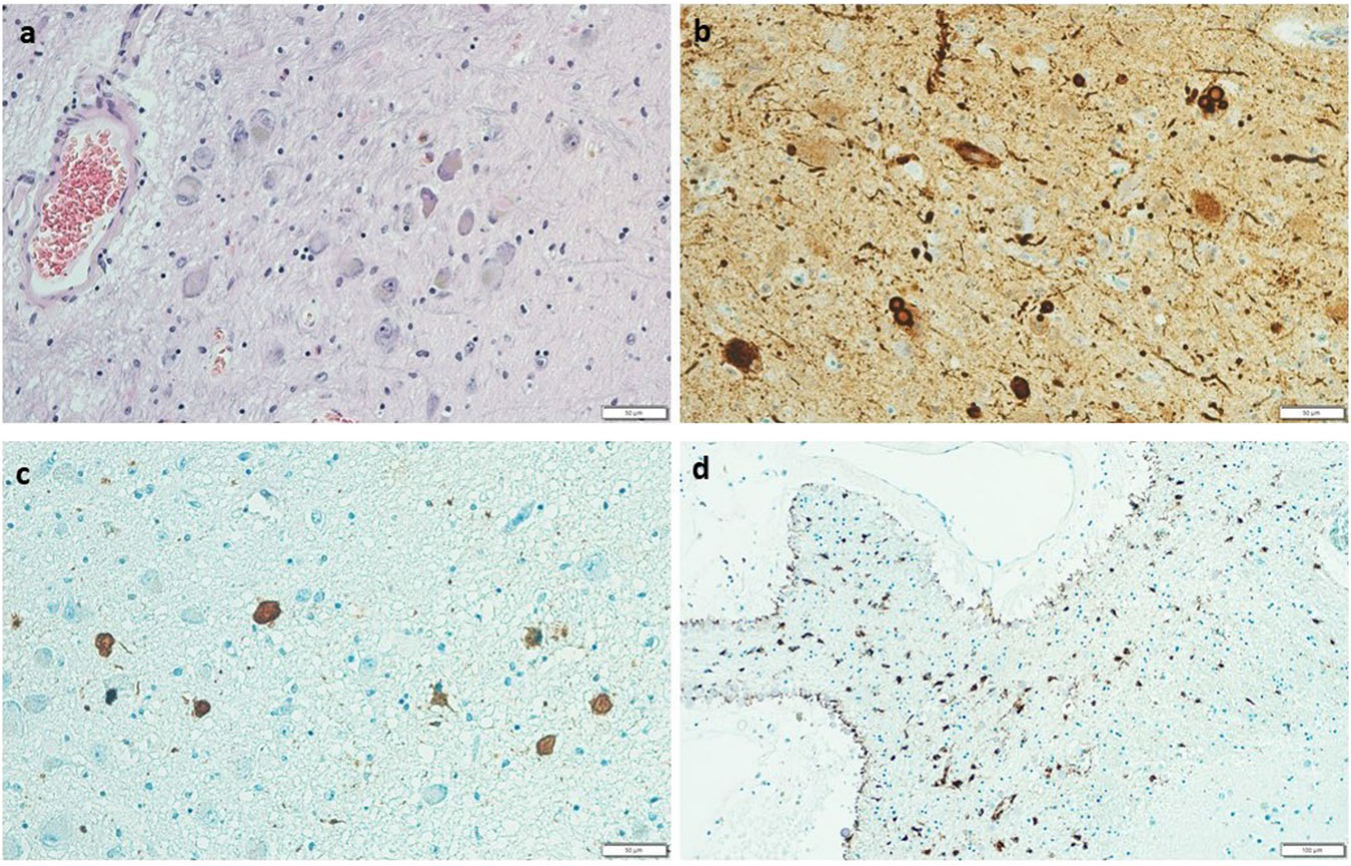
“Double-pathology“: α-synucleinopathy + tauopathy in Parkinson’s disease dementia (PDD) phenotype. **a** Lewy body and pale bodies in pontine raphe nucleus, HE staining, original magnification ×200. **b** Pathological deposits of α-synuclein in pontine raphe nucleus—Lewy bodies, granular cytoplasmic positivity, dystrophic neurites, and dots, stained with a monoclonal antibody against α-synuclein, original magnification ×200. **c** Pathological deposits of tau protein in pontine raphe nucleus—tangles, pretangles, grains, and threads, stained with a monoclonal antibody against hyperphosphorylated tau, original magnification ×200. **d** Pathological deposits of tau protein in basal ganglia associated with cribrous state—tau-astrogliopathy (ARTAG), stained with a monoclonal antibody against hyperphosphorylated tau, original magnification ×100.

**Fig. 3:**
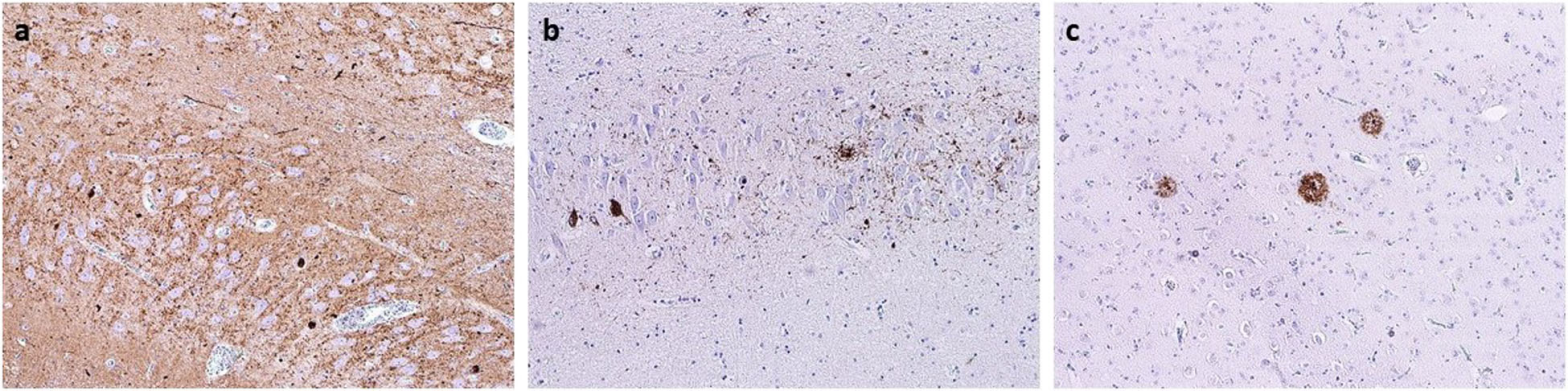
“Triple-pathology“: α-synucleinopathy + tauopathy + β-amyloid in progressive supranuclear palsy—parkinsonism (PSP-P) phenotype. **a** Pathological deposits of α-synuclein in the hippocampus—Lewy bodies, dystrophic neurites, and dots, stained with a monoclonal antibody against α-synuclein, original magnification ×100. **b** Pathological deposits of tau protein in the hippocampus—pretangles, threads, and grains, stained with a monoclonal antibody against hyperphosphorylated tau, original magnification ×100. **c** Amyloid plaques in hippocampus positive in immunohistochemical reaction with a monoclonal antibody against amyloid-β -peptide, original magnification ×100.

The trouble is that the pathology is not always “unique” in the sense of typical disease signs and hallmarks. Of course, the neuropathological examination in the typical “sporadic” PD will probably reveal only the pathology typical for the alpha-synucleinopathy (Fig. 1) and in typical PSP, the pathology typical for tauopathy^1,2^. However, in the majority of cases, the final neuropathological picture is more complicated than the ones just described. It is widely known, that the most frequently observed pathological finding in neurodegenerative disorders (including parkinsonism) is the “double-pathology” (Fig. 2) or even “triple-pathology” (Fig. 3)^3–8^, sometimes called “overlap syndrome”. It means that the characteristic “pure” pathological picture (Fig. 1) is a rather rare case, and that overlaps prevail (Figs. 2 and 3)^9,10^. Moreover, in many cases, the picture is complicated by concomitant vascular changes.

The question stands whether this observation is reflected intra vitam also in the clinical manifestations and whether the current clinical diagnostic criteria can serve as a valuable tool in the diagnostic process. It has been debated for more than the past 10 years that the “validated” and widely used clinical diagnostic criteria for some of the above-mentioned clinical entities are outdated and based on the state of knowledge at the time of their publication, i.e. the eighties or nineties of the past century^11–19^. Several groups are working hard on the establishment of the new criteria, namely for CBD, PSP, MSA and FTD, but the validation process will certainly take a few years.

The present work aims to re-appraise the issue of Lewy body (LB) diseases, based on past and contemporary nosological and neuropathological correlates and other molecular-biological aspects that may play a role in their pathogenesis^16,20–24^.

## The enigma of classifying PD and other LB disorders

Nowadays, the neurological community is facing a classification problem in the group of intraneuronal synucleinopathies, which covers the proper clinical diagnosis and differential diagnosis among PD, PDD, and DLB^5,25,26^.

### PD and its heterogeneity

Traditionally, sporadic (or idiopathic) PD has been recognized as a unique clinical entity, characterized by the presence of classical clinical signs, and by the typical pathology. The pathological hallmark of PD, the presence of LBs, was described by Friedrich Heinrich Lewy in his chapter of Max Lewandovsky’s neurology textbook, issued in Berlin in 1912. The disease-specific involvement of substantia nigra was then described by Tretiakoff in 1919, who also confirmed the existence of LBs (and named them after Lewy). The fact, that LBs are formed predominantly by pathological alpha-synuclein (αSyn) was revealed by Spillantini and colleagues only 80 years later^27,28^. The presence of this “alpha-synucleinopathy” was listed as the typical pathological feature of PD, either “sporadic” or “hereditary”^29^.

“Hereditary PD” is a roofing term, used for the Mendelian forms of PD. However, clinical manifestations of these hereditary disorders are not exactly those of “typical” PD, being in several cases rather suggestive of atypical parkinsonism. Currently, altogether 24 variants which are considered either to be “causal” for the development of parkinsonism or “associated” with its manifestation, were described^30–32^. These variants are listed in Table 1, together with a short description of the parkinsonian phenotype and morphological finding, typical for a given variant (Table 1). One can realize that from this list only 19 are “typical” phenotypes of the disease. The remaining five phenotypes rather resemble different variants of “atypical parkinsonism”^33^.

**Table 1:** Genes and cytogenetic locations in hereditary forms of Parkinson’s disease their clinical phenotypes and neuropathological findings.

Gene symbol	Cytogenetic location	Gene name	Inheritance	Phenotype	Pathology
PARK1	4q21–23	*SNCA*	AD	“Contursi kindred”, typical Parkinson’s disease manifesting with two or more cardinal signs (bradykinesia, rigidity, tremor, postural instability and with early or young onset (EOPD, YOPD); in some cases, the cognitive disturbance, psychotic signs, hyperreflexia, spasticity, and myoclonus were recorded	Severe degree of LB pathology. The majority of cases present also neurofibrillary tangles. TDP-43 pathology in the hippocampus in some cases
PARK2	6q25.2–q27	Parkin	AR	Typical Parkinson’s disease manifesting with two or more cardinal signs (bradykinesia, rigidity, tremor, postural instability) and with the early onset (EOPD); hyperreflexia, feet dystonia, and prominent retropulsion and sensory axonal neuropathy were recorded	LB pathology of different degrees present in a minority of cases; different degrees of tau inclusions and particularly typical PSP pathology present in part of the cases
PARK3	2p13	Unknown	AD	Typical Parkinson’s disease manifesting with two or more cardinal signs (bradykinesia, rigidity, tremor, postural instability) and with the late onset as seen in “sporadic” disease	Brain pathology is not known
PARK4	4q21–23	*SNCA*	AD	“Iowa kindred”, typical Parkinson’s disease manifesting with two or more cardinal signs (bradykinesia, rigidity, tremor, postural instability) with early or young onset (EOPD, YOPD); cognitive disturbance and dysautonomia were also recorded	Severe degree of LB pathology. The majority of cases present also neurofibrillary tangles. TDP-43 pathology in the hippocampus in some cases
PARK5	4p13	*UCHL1*	AD	Typical Parkinson’s disease manifesting with two or more of cardinal signs (bradykinesia, rigidity, tremor, postural instability) and with young onset (YOPD)	Brain pathology is not known
PARK6	1p35–36	*PINK1*	AR	Typical Parkinson’s disease manifesting with two or more of cardinal signs (bradykinesia, rigidity, tremor, postural instability) and with asymmetric early onset (EOPD); in some cases the dystonia, hyperreflexia and sleep benefit were recorded	Limited data (only one published case with typical LB pathology)
PARK7	1p36.23	*DJ1*	AR	Typical Parkinson’s disease manifesting with two or more of cardinal signs (bradykinesia, rigidity, tremor, postural instability) and with asymmetric early onset (EOPD); in some cases the loss of postural reflexes, bulbar signs, and muscle atrophy were recorded	Limited data (only one published case with typical LB pathology)
PARK8	12q12–13.1	*LRRK2*	AD	Typical Parkinson’s disease manifesting with two or more of cardinal signs (bradykinesia, rigidity, tremor, postural instability) and with late onset; in some cases, prominent lateralization of symptoms was recorded	LB pathology with significantly heterogeneous distribution; similarly heterogeneous spread of tau inclusions of different types, TDP-43 pathology in the hippocampus in a minority of cases
PARK9	1p36.13	*ATP13A2*	AR	Kufor–Rakeb syndrome, early-onset (EOPD) atypical parkinsonism manifesting with bradykinesia, rigidity, prominent hypomimia, spasticity, supranuclear gaze palsy, and dementia; in some cases, facial mini-myoclonus, oculogyris crises and dystonia were recorded	Limited data (only one published case in which LB pathology was absent)
PARK10	1p32	Unknown	Unclear	Typical Parkinson’s disease manifesting with two or more cardinal signs (bradykinesia, rigidity, tremor, postural instability) and with the late onset as seen in “sporadic” disease	Brain pathology is not known
PARK11	2q37.1	*GIGYF2*	AD	Typical Parkinson’s disease manifesting with two or more cardinal signs (bradykinesia, rigidity, tremor, postural instability) and with the late onset as seen in “sporadic” disease	Brain pathology is not known
PARK12	Xq21–q25	Unknown	X-linked	Typical Parkinson’s disease manifesting with two or more cardinal signs (bradykinesia, rigidity, tremor, postural instability) and with the late onset as seen in “sporadic” disease	Brain pathology is not known
PARK13	2p13.1	*HTRA2*	AD	Typical Parkinson’s disease manifesting with two or more cardinal signs (bradykinesia, rigidity, tremor, postural instability) and with the late onset as seen in “sporadic” disease; with extremely positive and sustained response to L-DOPA therapy	Brain pathology is not known
PARK14	22q13.1	*PLA2G6*	AR	Atypical early-onset parkinsonian syndrome (young adulthood), with dominant tremor, bradykinesia, and rigidity, other features included rapid cognitive decline, dysarthria, supranuclear gaze palsy, eyelid opening apraxia, hyperreflexia and spasticity (PSP-like phenotype)	LB pathology of different degrees; in majority of cases were also neurofibrillary tangles present. Presence of iron accumulation
PARK15	22q12.3	*FBX07*	AR	Atypical early-onset parkinsonian syndrome with very slow progression, dominant bradykinesia and rigidity in most of cases accompanied by spasticity and hyperreflexia; the presence of Babinski sign and dystonia has been recorded. Atypical parkinsonism with PSP-P phenotype	Limited data (only one published case, in which the LB pathology with minor Alzheimer-type changes was present)^a^
PARK16	1q32	Unknown	Unclear	Typical Parkinson’s disease manifesting with two or more cardinal signs (resting tremor, bradykinesia, rigidity, postural instability) with the late onset as seen in “sporadic” disease and with more rapid motor progression	Brain pathology is not known
PARK17	16q11.2	*VPS35*	AD	Typical Parkinson’s disease manifesting with two or more cardinal signs (dominant resting tremor, bradykinesia, rigidity, postural instability) and with the late onset as seen in “sporadic” disease; cramps were present in some pedigrees Atypical parkinsonism with PSP-P phenotype	Limited data (only two published cases; in one case was the LB pathology absent, in other case was the LB pathology with minor Alzheimer-type changes present)^a^
PARK18	3q27.1	*EIF4G1*	AD	Typical late-onset Parkinson’s disease, asymmetric manifestation with resting tremor, bradykinesia, rigidity, postural instability, and with the long and mild progression	Brain pathology is not known
PARK19A/B	1p31.3	*DNAJC6*	AR	A: atypical juvenile-onset parkinsonism with dominant akinesia and rigidity, postural instability, dysarthria, dystonia, pyramidal signs, and occasional epileptic seizures and mental retardation B: typical early-onset Parkinson’s disease with resting tremor, bradykinesia, rigidity, and good response do L-DOPA	Brain pathology is not known
PARK20	21q22.1	*SYNJ1*	AR	Atypical early-onset parkinsonism with dominant akinesia and rigidity, postural instability, shuffling gait, supranuclear gaze palsy, apraxia of eyelid opening, staring gaze, dysarthria, dystonia, and cognitive decline	Brain pathology is not known
PARK21	20p13	*TMEM230*	AD	Typical Parkinson’s disease manifesting with resting tremor, bradykinesia, and rigidity; with the asymmetric onset and sustained response to L-DOPA therapy	Typical LB pathology, in some cases, were the tau inclusions typical for PSP present
PARK22	7p11.2	*CHCHD2*	AD	Typical Parkinson’s disease with bradykinesia, rigidity, and gait disturbance; with the asymmetric onset and sustained response to L-DOPA therapy	Brain pathology is not known
PARK23	15q22.2	*VPS13C*	AR	Atypical early-onset parkinsonism with dominant akinesia and rigidity, postural instability, early cognitive decline, dysautonomia, axial symptoms, pyramidal signs, and dysautonomia	Limited data (only one published case, in which typical LB pathology together with neurofibrillary tangles were present)
PARK24	10q22.1	*PSAP*	AD	Typical adult-onset Parkinson’s disease with resting tremor, bradykinesia and rigidity; asymmetric onset and sustained response to L-DOPA therapy	Brain pathology is not known

*AD* autosomal dominant, *AR* autosomal recessive, *SNCA* Synuclein Alpha, *LRRK2* Leucin Rich Repeat Kinase2, *PINK1* PTEN-Induced Putative Kinase 1, *UCHL1* Ubiquitin Carboxyl-Terminal Hydrolase Isozyme L1, *ATP13A2* ATPase 13A2, *GIGYF2* GRB10 interacting GYF protein 2, *HTRA2* Htr A serine peptidase 2, *PLA2G6* phospholipase A2 group VI, *FBX07* F-box protein 7, *VPS35* VPS35 retromer complex component, *EIF4G1* eukaryotic translation initiation factor, *DNAJC6* DnaJ heat shock protein family (Hsp40), *SYNJ1* synaptojanin 1, *TMEM230* transmembrane protein 230, *CHCHD2* coiled-coil–helix–coiled-coil–helix domain containing 2, *VPS13C* vacuolar protein sorting 13 homologue C, *EOPD* early-onset Parkinson’s disease (onset at the age <40 years according to the EPDA definition), *YOPD* young-onset Parkinson’s disease (onset at the age <50 years according to the APDA definition), *PSP* progressive supranuclear palsy, *MSA* multiple system atrophy. The gene names and cytogenetic locations correspond to the current data in the OMIM database (www.omim.org).

^a^Mensikova et al.^58^.

Among these mendelian forms of hereditary parkinsonism is also the parkinsonism with the “typical” phenotype, listed under the gene name *LRRK2*. Despite the phenotypic homogeneity of *LRRK2* parkinsonism, 6 types of pathological findings have been reported in association with this phenotype so far. These included nigral LBs or diffuse LBs, nigral tau without LBs, Alzheimer’s type pathology, axonal spheroids, and degeneration of the zona compacta without LBs, tau or beta-amyloid (Aβ)^34^. Does it mean, that those people, who suffer from the *LRRK2* variant-induced disease, do not suffer from PD? Or, is the definition of PD, as defined by the current diagnostic criteria, outdated?

The fact that the same genetic variation may cause disparate clinical manifestations and pathological findings demonstrate a complex interplay of genetic, environment, and exposures. On the other side, there is a broad spectrum of clinical manifestations of “typical” Lewy-related brain pathology (Lewy pathology, LP) associated with various rare genetic abnormalities^35–37^, where a similar combination of factors can be assumed.

### Blurred differences between PDD and DLB

In some PD patients, cognitive impairment may be present. These cases were in the last decade separated into the newly established category of PDD^38^. Interestingly, these patients who manifested typical signs of PD together with cognitive impairment leading to overt dementia had also a different pathological correlate. This was the “typical” LB pathology accompanied by the presence of Aβ deposits in the limbic system^25^.

It is even more interesting when DLB is also considered. Here the amount of Aβ deposits in the brain should be much higher than in the PDD. The degree of Alzheimer’s pathology, its magnitude within the brain tissue, and the presence of cerebral amyloid angiopathy is probably the most significant pathological difference between these two phenotypes, PDD and DLB^5,10,39–41^. Nevertheless, there is no sharp pathological border between these two pictures, it is a smooth transition from the “pure” PD Lewy pathology to the “mixed” DLB pathology.

This morphological course has also another dimension; the extent of the above-mentioned changes within the brain structures. The concept of the specific spread of LB pathology and its clinical correlates, i.e. the premotor stage of PD, the motor stage of PD, and the subsequent development of cognitive deficit, was described by Braak and confirmed in further clinicopathological studies^8,42–48^. However, the results of other studies have shown that the severity of clinical symptoms, the duration of the disease, and the presence of cognitive decline or visual hallucinations do not correlate with the density of LBs^49,50^. In some patients with PDD, virtually no LBs were seen in the cortical regions or even outside the brainstem^45^. The question thus remains how the time of development of cognitive deficit and its severity is influenced by the extent and severity of concomitant AD pathology.

### Pitfalls of current classification in clinical practice

From the clinician’s point of view, the clinico-pathological correlation has its unique sense in fostering the recognition and supporting further research of that specific personalized (targeted) treatment. Nonetheless, its usefulness in routine clinical practice is rather limited. For the taxonomic classification of the disease or clinical syndrome and the *state of the art* clinical management, the disease should be coded according to the international standards, either ICD or DSM manuals; the same counts also for the recruitment of the patients into clinical trials.

In many neurological patients, particularly those suffering from neurodegenerative disease, this might represent a serious problem. In the initial (and even in the advanced) stages the typical signs of a given disease may not be present, or vice versa, phenotypic signs of another proteinopathy may be seen^34^. For this reason, often complex clinical diagnostic criteria are used. Still, their use is suggested in both scientific and clinical communities, and the diagnosis made on criteria is fully accepted in both the scientific and clinical environments.

## Are the clinical diagnostic criteria really useful?

### Deficiency of Parkinson’s Disease Society Brain Bank Criteria (UK-PDSBB)

The clinical diagnosis of PD has been established in the past 30 years mainly based on the UK-PDSBB, first proposed in 1988^51^. These were later validated in two clinico-pathological studies carried out at Queen Square, in Hughes’ original study in 1992 and its replication published in 2001 and 2002^11,52,53^. The criteria were created using the clinical notes and data retrieved from general practitioner (GP) files and their retrospective correlation with pathological findings. From today’s point of view, it is therefore questionable, whether these criteria will resist the light of today’s molecular genetics and molecular biology state of the art.

The key players in this field are currently the (permanently increasing) numbers of gene variants, causal or associated with the manifestation of “typical“ PD, combined with epigenetic factors, and Braak’s concept formulated almost 20 years ago^42,54,55^. Nevertheless, the”hotspot“ should be the observations that the pathological neurodegenerative process might manifest in a quite different way than usually described and known (Table 2)^15,35,56–59^.

**Table 2:** Arguments against the current concept of Lewy body diseases.

Current nosological concept	Arguments against current nosological concept	Arguments against Lewy bodies as key players in the pathological process of the Lewy body disease spectrum
Parkinson’s disease “sporadic” and “hereditary”	• loss of nigral neurons in other neurodegenerative diseases (i.e. PSP, MSA, SCA) • genes associated with LB pathology, but not with PD syndrome (i.e. *PLA2G6*, *FBXO7*, *DNAJC6*, *SYNJ1*, *VPS13C*, *C19ORF12*); the phenotype resembles “atypical” parkinsonism • genes clinically associated with PD, but not always with LB pathology (i.e. *LRRK2*, Parkin) • genes associated with both PD syndrome and LB pathology (i.e. S*NCA, GBA*), but in most cases were not pure LB pathology, as tau inclusions were frequent. • other non-PD syndromes with PD-like pathology (i.e. 22q deletion syndrome, *RAB39B* mutation, SCA2)	• the severity of clinical symptoms, disease duration, and presence of cognitive decline or visual hallucinations do not correlate with LBs density • the spread and localization of LB pathology is not identical to the localization and spread of αSyn pathology, as determined by semi-quantitative evaluation of LBs in large autopsy series • the cell loss has been shown to precede the formation of LBs • Lewy body is not composed only by αSyn aggregates Mechanisms considered • the effect of concomitant (particularly AD) pathology as such or synergistic relationship between AD and αSyn pathology leading to hyperphosphorylation and subsequent αSyn aggregation • αSyn oligomers preceding the formation of LBs can mediate cell damage and later lead to a further aggregation • different αSyn strains differing in conformational properties that exhibit different cell toxicity and differences in the ability to induce tau protein aggregation • selective vulnerability due to the anatomical configuration of neurons, predisposing to early axonal involvement; αSyn aggregation starts in the axonal compartment and progresses back towards the cell body, axons become dystrophic with alterations in axonal transport, and this leads to cell death • synaptic dysfunction due to presynaptic αSyn microaggregates that impair vesicle trafficking and neurotransmitter release leading to postsynaptic dendritic spines degeneration and loss of synaptic connections • genetic factors leading to lysosomal dysfunction (i.e. *GBA*, *SCARB2* and other cellular alterations that remain to be elucidated
UK-PDSBB clinical diagnostic criteria (Gibb et al.^51^) • Bradykinesia AND at least one of the following • Muscular rigidity • 4-6 Hz rest tremor • Postural instability AND three or more of supportive prospective positive criteria		
Current pathological criteria of Parkinson’s disease (Braak et al.^42^) • Neuronal loss in substantia nigra and presence of Lewy body pathology		
PDD and DLB		
PDD clinical diagnostic criteria (Emre et al.^38^) DLB clinical diagnostic criteria (McKeith et al.^67^) Clinically • shared core features (dementia, cognitive fluctuations, and visual hallucinations) in the setting of overt or latent parkinsonism	• insufficient clinical data and inconsistent pathological techniques of cerebral autopsies in patient sets used for meta-analysis in the formulation of PDD clinical diagnostic criteria • 25% DLB patients never develop parkinsonian symptoms leading to a misdiagnosis of AD • it is not clear to what extent AD-related lesions may contribute to the timing of the dementia onset relative to motor signs • The question whether the “1-year rule” is a biologically valid distinction, or whether they are merely subtypes in a continuum of LBDs	
Pathologically • phased widespread cortical and subcortical α-synuclein deposits—Lewy pathology (Lewy bodies and Lewy neurites) • +/− β-amyloid and tau pathologies in both entities		
The diagnosis is based on an arbitrary distinction between the time of onset of motor and cognitive symptoms (1-year rule)		

*UK-PDSBB* United Kingdom Parkinson’s Disease Society Brain Bank, *PD* Parkinson’s disease, *SNpc* substantia nigra pars compacta, *PDD* Parkinson’s disease dementia, *DLB* dementia with Lewy bodies, *PSP* progressive supranuclear palsy, *MSA* multiple system atrophy, *SCA* spinocerebellar ataxia, *LB* Lewy body/ies, *PLA2G6*, *C19ORF12*, *FBXO7*, *DNAJC6*, *SYNJ1*, *VPS13C*, S*NCA*, *GBA*, *RAB39B*, *SCARB2* names of hereditary Parkinson’s disease genes, *AD* Alzheimer’s disease, *αSyn* alpha-synuclein.

A deeper insight into the structure of UK-PDBB criteria will reveal that they are only the diagnostic criteria of the parkinsonian syndrome (in their Step 1). Parts Step 2 and Step 3 containing exclusion and supportive positive criteria are full of non-specific, frequently obsolete signs based on retrospective data, derived from often incomplete GP clinical files stored in the London Brain Bank together with the fixed brains. However, the American attempt to create an “upgrade” of UK-PDSBB criteria (named NINDS-PD criteria) was only rarely cited and never reached the level of routine clinical use^60,61^. So, there are enough relevant reasons to put into discussion the reliability of the 34-years old UK-PDSBB clinical diagnostic criteria. They are still used not only for the confirmation of clinical diagnosis when the patients are recruited into the clinical trials but also as a universal tool for any clinical and clinico-pathological research in PD.

### An insufficient mainstay for the formulation of PDD criteria

PDD as a novel “subtype” of the LBD has been gradually recognized in the nineties of the last century. The principal reason which led to its identification was—without any doubt—the introduction of novel drugs into the PD treatment armamentarium, hand-in-hand with the introduction of the treatment of late, advanced, and complicated PD, i.e. deep brain stimulation and subcutaneous apomorphine infusions. Both these approaches led to substantially longer survival of PD patients, so the cases with manifest dementia appeared. In other words, dementia related to PD was unmasked.

When Emre in 2003 discussed the concept of PDD on a more extensive basis, he introduced two most important risk factors: older age as such, and older age at the moment of motor symptoms manifestation^62^. The detailed description of typical PDD phenotype together with the first suggestion of clinical diagnostic criteria was published in 2007. International experts, led by Emre, indeed performed a critical meta-analysis of published studies. They particularly extracted the neuropsychological manifestations of the typical PDD cognitive disorder, i.e. the progressive executive dysfunction, only later accompanied by the general cognitive dysfunction. They also summarized the results of 24 clinical–pathological studies, published in 1979–2005^38^.

This meta-analysis has two weak points (Table 2). The first is the fact, that the presence of LB pathology has been in the examined studies assessed in three different ways. The second is the fact that all clinical–pathological studies were done retrospectively using brain bank specimens, while the quality of donors’ clinical files was not—and still is not—known (as a model of the accuracy of clinical data may serve for instance the paper by Guo et al.^63^). Therefore, it is not clear on the basis of which criteria the diagnosis of cognitive impairment was determined. Nevertheless, the whole concept of PDD clinical existence still stands on that paper. The evidence that the neuropsychological profile of DLB and PDD practically does not differ, notably in the initial phase, has become apparent in the past few years (Table 2)^61,64^.

Another noteworthy aspect of this meta-analysis is the fact, that until the discovery of ubiquitin staining, the dementia was attributed to s.c. “Alzheimer’s pathology” of examined brains. After the introduction of ubiquitin staining, dementia was attributed to the “pathological changes of heterogenous origin”, including “Alzheimer’s and vascular”. Only after 1997, i.e. after the discovery of αSyn and the introduction of routine examination of its presence were the cases of parkinsonism accompanied by cognitive deficit attributed to the progressive “Lewy-body pathology”.

It is also important to mention, that from the strict pathological point of view, there is practically no difference between PD and PDD. Even the experienced neuropathologist is not able to differentiate between these two “disorders”, being able only to recognize the degree of αSyn deposit progression, distribution, and density. Considering all the above-mentioned facts, it is highly questionable for which disorder have Emre and colleagues established their clinical diagnostic criteria in 2007.

### The last version of DLB criteria suggests the use of the general term LBD

The next subtype of LBD is the DLB phenotype. In contrast to PDD, this phenotype is a bit better “bordered”, its definition is more intelligible and the pathological finding is unique. This is characterized by diffuse alpha-synucleinopathy, accompanied in most cases by Alzheimer’s changes, especially senile plaques.

The birth of the DLB as a nosological entity was complicated and took many years of scientific debates, consensus meetings, publications, which ran continuously for almost the last decade of the 20th century. Finally, the existence of pathological co-habitation of Alzheimer’s pathological changes together with the diffuse appearance of LBs led the expert panel to the opinion, that the former attempts to name this disease were always the attempts to describe the findings typical for DLB.

The first clinical diagnostic criteria were published by McKeith et al. in 1996; the revised version came in 2005 and the last revision is from 2017^65–67^. In the last revision, the nosological entity DLB has been classified rather as “one of the phenotypes in the broader spectrum of LBD”.

According to the 2017 clinical diagnostic criteria, for the diagnosis of “probable” DLB is necessary the presence of dementia and other neuropsychological signs as the fluctuations of cognitive dysfunction and fluctuations of awareness and wakefulness, recurrent visual hallucinations, REM sleep behavioural disorder, and one or more spontaneously manifested parkinsonian motor signs. It is known that, while the parkinsonian motor symptoms are present in 25–50% of patients at the moment when the diagnosis of DLB is made, in other cases motor signs developed in the course of the disease. However, almost one-fourth of patients will never manifest any parkinsonian motor signs (Table 2). It is undoubtedly important to highlight this point. In many clinical-pathological studies, the absence of motor signs was the principal cause of diagnostic errors in cases, which were later pathologically diagnosed as DLB^68–70^. The original idea, that the parkinsonian signs appear in DLB patients shortly before the onset of cognitive and psychiatric disorder, and that they are usually rather mild, was substantially revised. Nowadays, the prevailing opinion is that the parkinsonian signs develop only after the manifestation of cognitive disorders and that they can progress into the severity similar to advanced PD. So, the clinical diagnosis of DLB is in majority of cases a diagnosis “per exclusionem”^18,70–72^.

The different phenotypes of LBD are in both clinical routine and research classified based on the mutual relationship between parkinsonian motor signs and the signs of cognitive dysfunction. The “one-year rule” has been established already in the first version of McKeith criteria^65^. It has been arbitrarily determined, that “*if the cognitive disorder appears up to 12 months following the manifestation of parkinsonian signs, the DLB should be considered rather than PDD, no matter what is the character of cognitive disorder*”. On the contrary, if the parkinsonian signs are present at the moment of cognitive disorder manifestation for a period longer than 12 months, the diagnosis should strongly incline towards PDD (Table 2). However, as was already mentioned, up to 25% of patients do not manifest motor symptoms^68,71^.

The presence of at least one parkinsonian sign is among the “core clinical features” of the newly established disorder that has been named “MCI-LB” and that represents the initial phase of DLB^19^. However, there is no substantial difference between the initial manifestation of cognitive dysfunction in PDD and DLB. So, it may be rather said, that the cognitive dysfunction which appears in a patient suffering from parkinsonian motor signs, and its gradual progression usually lead to the reconsideration of the original PD diagnosis to PDD or DLB. The clinical differentiation between PDD and DLB is possible only gradually (if possible at all), and is based on the appearance of the severity and speed of cognitive dysfunction progression, its fluctuations, and the presence of pathognomonic visual hallucinations. The last version of the DLB clinical diagnostic criteria dealt with a complicated situation by the final statement: “*DLB should be diagnosed when dementia occurs before or concurrently with parkinsonism. The term PDD should be used to describe dementia that occurs in the context of well-established PD. In a practice setting the term that is most appropriate to the clinical situation should be used and generic terms such as LBD are often helpful*”^67^.

## Pathogenic mechanisms beyond LBs or LBs as an indirect indicator of this disease spectrum

As follows from the previous consideration, the current concept behind classification within this disease spectrum is still based on retrospective clinicopathological studies, which focused exclusively on the presence of LBs and their clinicopathological relevance. However, since the initial description of LBs as a pathological hallmark of PD and the formulation of Braak’s concept of the specific spread of LB pathology, evidence has been accumulating that not only LBs (and their density and distribution) are key players in this group of diseases^73^. So, what is the true significance of LBs in the pathogenesis of this disease spectrum, and what are the other biological relationships between the entities for which LBs are a common link?

Given the growing knowledge in the field of cell and molecular biology and molecular genetics, it seems that LBs as such do not play a major role in the pathological process and are rather an indirect indicator of these diseases. The spectrum of αSyn accumulations in LB disorders is much broader than the mere presence of LBs and involves also depositions in synapses and neurites^74–76^. The use of modern techniques has revealed further pathological features including the presence of concomitant pathology as such or synergistic relationship between concomitant and αSyn pathology, selective vulnerability due to the anatomical configuration of neurons, synaptic dysfunction, and role of genetic factors^77^. Unfortunately, documentation of most of these aspects is lacking in the majority of existing clinicopathological studies (Table 2).

### Combined pathologies

One of the factors that may be behind the development of cognitive deficit in addition to LBs is the parallel presence of AD pathology. The combination of LBs and AD pathology predicts dementia in PD much better than the severity of any single pathology^3^. In clinical studies in patients with newly diagnosed PD, the cerebrospinal fluid (CSF) biomarker evidence for Aβ pathology was a significant predictor of subsequent cognitive impairment^3,78^. Similarly, other studies comparing patterns of CSF biomarkers between patients with DLB and PDD showed that lower levels of Aβ1-42 (combined with higher tau levels) are associated with DLB rather than PDD and are seen particularly in patients with more rapidly progressive dementia^79,80^.

### The degree of α-synuclein phosphorylation due to the synergistic effect of AD pathology

Several in vivo and animal studies have shown a strong correlation between the extent of neurofibrillary tangles, neuritic plaques, and αSyn, suggesting synergistic effects of AD and αSyn pathology^81,82^.

Phosphorylation is considered as a potential mechanism for this synergy^83^. In experimental studies, recombinant Aβ can induce phosphorylation of αSyn at Ser129, which is considered to be a major modifier of αSyn in PDD/DLB^84,85^. Whereas only a small fraction of αSyn (<4%) is phosphorylated in healthy brains, a dramatic accumulation of pS129 (>90%) has been observed within LBs. These findings suggest that this posttranslational modification may play an important role in the regulation of αSyn aggregation, LBs formation, and neuronal degeneration. Higher levels of phosphorylated αSyn are present in the early stages of PDD/DLB before the occurrence of LB pathology, and levels of phosphorylated αSyn correlate with disease severity^86,87^.

### Synuclein oligomers-induced cell loss precedes the formation of LBs

As was already mentioned, LBs considered to be the pathological hallmark of this group of diseases may not even play any causal role in their pathophysiology. As has been shown, the severity of clinical symptoms, disease duration, and presence of cognitive decline or visual hallucinations do not correlate with LBs density^49,50^. In some patients with PDD, virtually no LBs were seen in cortical regions or even outside the brainstem^88^. Furthermore, it has been shown, that cell loss can precede LB accumulation, calling into question the hypothesis that LBs are the toxic agents in PD which drive neurodegeneration^89,90^.

This role should be rather attributed to LBs precursors called oligomers (which cannot be seen in the light microscope) than to the LBs themselves. There is evidence that initial amorphous αSyn deposits known as “pale bodies” and “pale neurites” can mediate cell damage and later lead to a further aggregation^91^. Thus, neurodegeneration and cell death do not appear to be caused by LBs, but LBs rather protect “toxic” αSyn aggregates. LBs and Lewy neurites are thus more probably an indirect indicator of the disease stage and not a reflection of the whole extent of the neurodegenerative process^92,93^.

### Different conformational properties of α-synuclein

Another aspect supporting the fact that LBs are not the key players in neurodegeneration is the description of different αSyn strains differing in conformational properties. These strains exhibit different cell toxicity and differences in the ability to induce tau protein aggregation. This is again a situation where pathological processes preceding the formation of LBs may affect the course and progression of the disease. Thus, different αSyn strains may also be the factor involved in the phenotypic variability of this group of diseases^94,95^. It is probable that as yet unknown genetic factors will apply here.

### Selective vulnerability due to anatomical configuration of neurons

The selective vulnerability of specific neuronal populations is considered to be one of the factors involved in the specific distribution of pathological changes and the resulting clinical phenotype. The anatomical configuration of neurons (especially those with long hyperbranched axons that project widely to innervate multiple brain regions) is thought to be one of its causes^77^. More recent neuro-histological studies support the theory that axonal involvement is critical. αSyn aggregation starts in the axonal compartment and progresses back towards the cell body, axons become dystrophic with alterations in axonal transport, and this ultimately leads to cell death^96,97^.

It has been shown in PD cases that loss of dopamine is more profound at the axon terminals in the caudate and putamen than is the loss of nigral neurons; it suggests that degeneration is greatest in distal parts of the cell. Other neurons preferentially affected in PD, PDD, and DLB also show a similar anatomical configuration. These are mainly cholinergic cells of the nucleus basalis of Meynert that are strongly implicated in the pathogenesis of dementia in PD^98^ or serotonergic cells of the raphe nucleus, which also have extensive axon projections^99^. Similarly, the long unmyelinated axons of the peripheral autonomic nervous system may explain the early and prominent involvement of autonomic symptoms in both DLB and PD.

### Synaptic dysfunction

The synapse is another potential location for early involvement. It seems, that presynaptic involvement is an event that precedes neuronal death. Presynaptic αSyn microaggregates can easily impact post-synaptic dendritic spines. Almost complete loss of the dendritic spines in frontal cortical neurons has been found in patients with DLB compared with age-matched controls using visualization of silver impregnation technique. A similar loss of dendritic spines was seen in the striatum in PD^100,101^. αSyn aggregation starts at either the synapse or axon branch points that subsequently affect vesicle trafficking and impair neurotransmitter release. This causes postsynaptic dendritic spines degeneration with loss of synaptic connections^93^.

### Role of genetic factors

In contrast to PD (and therefore also PDD) in which numerous disease-related gene loci have been described, it has long appeared in DLB that genetic factors play here virtually no role. Only in the last decade, some variants have been identified in DLB. One of the first hints that genetics plays the same role in PD and DLB came from the studies of glucocerebrosidase (*GBA*). Homozygous *GBA* mutations are known to cause a lysosomal storage disorder (Gaucher disease), while heterozygous mutations are considered a risk factor for PD^102^. In DLB, a similar effect of these mutations was identified suggesting that there is an identical underlying lysosomal dysfunction present in both diseases^103^.

An association study showed that common variability is also involved in DLB. Variants at the *APOE*, *SNCA*, and *SCARB2* loci were shown to be associated with DLB cases^104^. While the association of *APOE* variants was identical to that observed in AD, the *SNCA* and *SCARB2* variants have different association profiles than the associations reported for the same loci in PD. Since DLB is not only characterized by LBs but also by the presence of Aβ, the association of DLB with the ε4 allele of *APOE* is likely driven by the Aβ pathology-promoting effect of this particular variant. Regarding the *SNCA* gene, the haplotype conferring risk is different for PD and DLB; in PD having an association with 3′ of gene and DLB appearing to occur 5′ of the gene. Although it is not clear at this stage what are the implications of this difference, it may influence the distribution of the LBs in the brain tissue, presumably through differential expression of the gene. The *SCARB2* gene encodes lysosomal protein that is associated with PD, but unlike PD where it is not considered a major risk factor, with DLB its risk seems relatively high^104^. These data indicate that DLB has not only a genetic component but also that this component has a unique architecture (when compared to PD and AD) leading to the specific phenotype of DLB.

The genetic differences between PDD and DLB have, so far, not been studied in detail. Some factors predisposing to the development of earlier dementia in PDD cases have a genetic basis. For example, rapid eye movement sleep behaviour disorder, which is predictive of cognitive involvement when it occurs in patients with PD^105^, is more common in patients carrying *GBA* and *SNCA* mutations and less common in patients carrying *LRRK2* mutations^73^.

## Conclusions

In our opinion, the current pieces of knowledge suggest that PD, PDD, and DLB represent closely related but different, heterogeneous subtypes of an α-synuclein-associated disease spectrum. Given the controversies about the nosology of these disorders, continuous effort is necessary to distinguish among them more clearly and to clarify the underlying pathogenic mechanisms to enable effective mechanistic-based treatment, considering that no disease-modifying therapies are currently available. Further elucidation of the relations between PD, PDD, and DLB including better insight into common genetic and epigenetic risk factors and pathogenetic molecular pathways responsible for the clinical manifestations of these disorders will be necessary as the basis for future preventive and symptomatic treatment options.

Clarification and understanding of biological factors will most likely lead to a shift in the concepts of these diseases and to thinking about their natural course from a pathobiological point of view. In the era of personalized medicine, the genetic risk may be used in early recognition to predict the risk of developing cognitive deficit in patients with PD pathology and may replace the current arbitrary clinical criteria still used to distinguish DLB from the questionable concept of PDD. The understanding of synucleinopathies nosology may be the best we have to proceed with clinical trials. Nevertheless, it must be kept in mind that the artificial differentiation instead of “aggregation” of these entities may be the reason why these trials frequently fail.

Returning to the original question of whether to clinically label a group of these diseases as “Lewy body diseases” or “diseases with Lewy bodies”, the latter seems more accurate because of the current state of knowledge. Nevertheless, there should be only two: PD and DLB.

## Data Availability

Data sharing is not applicable to this article as no data sets were generated or analysed during the current study.

## References

[1] Arendt T, Stieler JT, Holzer M (2016). Tau and tauopathies. Brain Res. Bull..

[2] Kovacs GG (2019). Molecular pathology of neurodegenerative diseases: principles and practice. J. Clin. Pathol..

[3] Compta Y (2011). Lewy – and Alzheimer-type pathologies in Parkinson’s disease dementia: which is more important?. Brain.

[4] Kovacs GG (2013). Non-alzheimer neurodegenerative pathologies and their combinations are more frequent than commonly believed in the elderly brain: a community-based autopsy series. Acta Neuropathol..

[5] Jellinger KA (2018). Dementia with Lewy bodies and Parkinson’s disease dementia: current concepts and controversies. J. Neural Transm..

[6] Kovacs GG (2019). Are comorbidities compatible with a molecular pathological classification of neurodegenerative diseases?. Curr. Opin. Neurol..

[7] Das S, Zhang Z, Ang LC (2020). Clinicopathological overlap of neurodegenerative diseases: a comprehensive review. J. Clin. Neurosci..

[8] Geut H (2020). Neuropathological correlates of parkinsonian disorders in a large Dutch autopsy series. Acta Neuropathol. Commun..

[9] Galpern WR, Lang AE (2006). Interface between tauopathies and synucleinopathies: a tale fo two proteins. Ann. Neurol..

[10] Foguem C, Manckoundia P (2018). Lewy body disease: clinical and pathological “overlap syndrome“ between synducleinopathies (Parkinson disease) and tauopathies (Alzheimer disease). Curr. Neurol. Nerosci. Rep..

[11] Hughes AJ, Daniel SE, Lees AJ (2001). Improved accuracy of clinical diagnosis of Lewy body Parkinson’s disease. Neurology.

[12] Schrag A, Ben-Shlomo Y, Quinn N (2002). How valid is the clinical diagnosis of Parkinson’s disease in the community?. J. Neurol. Neurosurg. Psychiatry.

[13] Litvan I (2003). Movement Disorders Society Scientific Issues Committee report: SIC Task Force appraisal of clinical diagnostic criteria for Parkinsonian disorders. Mov. Disord..

[14] Postuma RB (2015). MDS clinical diagnostic crietria for Parkinson’s disease. Mov. Disord..

[15] Mensikova K, Tuckova L, Ehrmann J, Kanovsky P (2016). Unusual phenotype of pathologically confirmed progressive supranuclear palsy with autonomic dysfunction and cerebellar ataxia. Medicine.

[16] Höglinger G (2017). Clinical diagnosis of progresive supranuclear palsy: The Movement Disorders Society Criteria. Mov. Disord..

[17] Marsili L, Rizzo G, Colosimo C (2018). Diagnostic criteria for Parkinson’s disease: from James Parkinson to the concept of prodromal disease. Front. Neurol..

[18] Rizzo G (2018). Accuracy of clinical diagnosis of dementia with Lewy bodies: a systematic review and meta-analysis. J. Neurol. Neurosurg. Psychiatry.

[19] Mc Keith IG (2020). Research criteria for the diagnosis of prodromal dementia with Lewy bodies. Neurology.

[20] Armstrong MJ (2013). Criteria for the diagnosis of corticobasal degeneration. Neurology.

[21] Chare L (2014). New criteria for frontotemporal dementia syndromes: clinical and pathological diagnostic implications. J. Neurol. Neurosurg. Psychiatry.

[22] Höglinger GU, Respondek G, Kovacs GG (2018). New classification of tauopathies. Rev. Neurol..

[23] Respondek G, Levin J, Höglinger GU (2018). Progressive supranuclear palsy and multiple system atrophy: clinicopathological concepts and therapeutic challenges. Curr. Opin. Neurol..

[24] Jabbari E (2020). Diagnosis accross the spectrum of progressive supranuclear palsy and corticobasal syndrome. JAMA Neurol..

[25] McCann H, Stevens CH, Cartwright H, Halliday GM (2014). α-synucleinopathy phenotypes. Park. Relat. Disord..

[26] Caproni S, Colosimo C (2020). Diagnosis and differential diagnosis of Parkinson disease. Clin. Geriatr. Med..

[27] Spillantini MG (1997). Alpha-synuclein in Lewy bodies. Nature.

[28] Spillantini MG, Goedert M (2018). Neurodegeneration and the ordered assembly of α-synuclein. Cell Tissue Res..

[29] Riederer P (2019). α-synuclein in Parkinson’s disease: causal or bystander?. J. Neural Transm..

[30] Hernandez DG, Reed X, Singleton AB (2016). Genetics in Parkinson disease: Mendelian versusu non-Mendelian inheritance. J. Neurochem..

[31] Puschmann A (2017). New genes causing hereditary Parkinson’s disease or parkinsonism. Curr. Neurol. Neurosci. Rep..

[32] Singleton AB, Hardy J (2019). Progress in the genetic analysis of Parkinson’s disease. Hum. Mol. Gen..

[33] Weissbach A, Wittke C, Kasten M, Klein C (2019). “Atypical“ Parkinson’s disease – genetic. Int. Rev. Neurobiol..

[34] Tolosa E, Vila M, Klein C, Rascol O (2020). LRRK2 in Parkinson disease: challenges of clinical trials. Nat. Rev. Neurol..

[35] Wilson GR (2014). Mutations in RAB39B cause X-linked intellectual disability and early-onset Parkinson disease with alpha-synuclein pathology. Am. J. Med. Genet..

[36] Butcher NJ (2013). Association between early-onset Parkinson disease and 22q11.2 deletion syndrome: identification of a novel genetic form of Parkinson disease and its clinical implications. JAMA Neurol..

[37] Takao M (2011). Spinocerebellar ataxia type 2 is associated with Parkinsonism and Lewy body pathology. BMJ Case Rep..

[38] Emre M (2007). Clinical diagnostic criteria for dementia associated with Parkinson’s disease. Mov. Disord..

[39] Mori H (2005). Pathological substrate of dementia in Parkinson’s disease-its relation to DLB and DBLD. Parkinsonism Relat. Disord..

[40] Jellinger KA (2009). A critical evaluation of current staging of alpha-synuclein pathology in Lewy body disorders. Biochim. Biophys. Acta.

[41] Hansen DS (2021). Novel clinicopathological characteristics differentiate dementia with Lewy bodies from Parkinson’s disease dementia. Neuropathol. Appl. Neurobiol..

[42] Braak H (2003). Staging of brain pathology related to sporadic Parkinson’s disease. Neurobiol. Aging.

[43] Wakabayashi K (2020). Where and how alpha-synuclein pathology spreads in Parkinson’s disease. Neuropathology.

[44] Attems J (2021). Neuropathological consensus criteria for the evaluation of Lewy body pathology in post-mortem brains: a multi-centre study. Acta Neuropathol..

[45] Bove C, Travagli RA (2019). Neurophysiology of the brainstem in Parkinson’s disease. J. Neurophysiol..

[46] Keir LHM, Breen DP (2020). New awakenings: current understanding of sleep dysfunction and its treatment in Parkinson’s disease. J. Neurol..

[47] Tremblay C, Mei J, Frasnelli J (2020). Olfactory bulb surroundings can help to distinguish Parkinson’s disease from non-parkinsonian olfactory dysfunction. Neuroimage Clin..

[48] Adler CHH (2019). Unified staging system for Lewy body disorders: clinicopathologic correlations ond comparison to Braak staging. J. Neuropathol. Exp. Neurol..

[49] Mattila PM, Rinne JO, Helenius H, Dickson DW, Röyttä S (2000). Alpha-synuclein-immunoreactive cortical Lewy bodies are associated with cognitive impairment in Parkinson’s disease. Acta Neuropathol..

[50] Jellinger KA (2009). Formation and development of Lewy pathology: a critical update. J. Neurol..

[51] Gibb WRG, Lees AJ (1988). The relevance of Lewy body to the pathogenesis of idiopathic Parkinson’s disease. J. Neurol. Neurosurg. Psychiatry.

[52] Hughes AJ, Daniel SE, Kilford L, Lees AJ (1992). Accuracy of clinical diagnosis of idiopathic Parkinson’s disease: a clinico-pathological study of 100 cases. J. Neurol. Neurosurg. Psychiatry.

[53] Hughes AJ, Daniel SE, Ben-Shlomo Y, Lees AJ (2002). The accuracy of diagnosis of parkinsonian syndromes in a specialist movement disorder service. Brain.

[54] Braak H, Ghebremedhin E, Rüb U, Bratzke H, Del Tredici K (2004). Stages in the development of Parkinson’s disease-related pathology. Cell Tissue Res..

[55] Braak H (2006). Pathology associated with sporadic Parkinson’s disease-where does it end?. J. Neural Transm..

[56] Batla A (2013). Markedly asymmetric presentation in multiple system atrophy. Parkinsonism Relat. Disord..

[57] Mensikova K (2013). Progressive supranuclear palsy phenotype mimicking synucleinopathies. J. Neurol. Sci..

[58] Mensikova K (2019). Atypical parkinsonism of progressive supranuclear palsy-parkinsonism (PSP-P) phenotype with rare variants in FBXO7 and VPS35 genes associated with Lewy body pathology. Acta Neuropathol..

[59] Respondek G, Stamelou M, Höglinger GU (2019). Classification of atypical parkinsonism per pathology versus phenotype. Int. Rev. Neurobiol..

[60] Gelb DJ, Oliver E, Gilman S (1999). Diagnostic criteria for Parkinson’s disease. Arch. Neurol..

[61] Palermo G, Del Prete E, Bonuccelli U, Ceravolo R (2020). Early autonomic and cognitive dysfunction in PD, DLB and MSA: blurring the boundaries between α-synucleinopathies. J. Neurol..

[62] Emre M (2003). Dementia associated with Parkinson’s disease. Lancet Neurol..

[63] Guo L, Itaya M, Takanashi M, Mizuno Y, Mori H (2005). Relationship between Parkinson disease with dementia and dementia with Lewy bodies. Park. Relat. Disord..

[64] Aldridge G, Birnschein A, Denburg NL, Narayanan NS (2018). Parkinson’s disease dementia and dementia with Lewy bodies have similar neuropsychological profiles. Front. Neurol..

[65] McKeith IG (1996). Consensus guidelines for the clinical and pathologic diagnosis of dementia with Lewy bodies (DLB): report of the Consortium on DLB International Workshop. Neurology.

[66] McKeith IG (2005). Diagnosis and management of dementia with Lewy bodies: Third Report of the DLB Consortium. Neurology.

[67] McKeith IG (2017). Diagnosis and management of dementia with Lewy bodies. Fourth Consensus Report of the DLB Consortium. Neurology.

[68] Kaur B (2013). Extrapyramidal signs by dementia severity in Alzheimer disease and dementia with Lewy bodies. Alzheimer Dis. Assoc. Disord..

[69] Walker Z, Possin KL, Boeve BF, Aarsland D (2015). Lewy bodies dementias. Lancet.

[70] Outeiro TF (2019). Dementia with Lewy bodies: an update and outlook. Mol. Neurodegener..

[71] Donaghy PC, McKeith IG (2014). The clinical characteristics of dementia with Lewy bodies and a consideration of prodromal diagnosis. Alzheimers Res. Ther..

[72] Gomperts SN (2016). Lewy body dementias: dementia with Lewy bodies and Parkinson disease dementia. Continuum.

[73] Weil RS, Lashley TL, Bras J, Schrag AE, Schott JM (2017). Current concepts and controversies in the pathogenesis of Parkinson’s disease dementia and dementia with Lewy bodies. F1000Res.

[74] Schulz-Schaeffer WJ (2010). The synaptic pathology of alpha-synuclein aggregation in dementia with Lewy bodies, Parkinson’s disease and Parkinson’s disease dementia. Acta Neuropathol..

[75] Rohan Z, Milenkovic I, Lutz MI, Matej R, Kovacs GG (2016). Shared and distinct patterns of oligodendroglial response in synucleinopathies and tauopathies. J. Neuropathol. Exp. Neurol..

[76] Colom-Cadena M (2017). Synaptic phosphorylated alpha-synuclein in dementia with Lewy bodies. Brain.

[77] Roberts RF, Wade-Martins R, Alegre-Abarrategui J (2015). Direct visualization of alpha-synuclein oligomers reveals previously undetected pathology in Parkinson’s disease brain. Brain.

[78] Postuma RB (2016). Abolishing the 1-year rule: how much evidence will be enough?. Mov. Disord..

[79] van Steenoven I (2016). Cerebrospinal fluid Alzheimer’s disease biomarkers across the spectrum of Lewy body diseases: results from a large multicenter cohort. J. Alzheimers Dis..

[80] Andersson M, Zetterberg H, Minthon L, Blennow K, Londos E (2011). The cognitive profile and CSF biomarkers in dementia with Lewy bodies and Parkinson’s disease dementia. Int. J. Geriatr. Psychiatry.

[81] Irwin DJ (2017). Neuropathological and genetic correlates of survival and dementia onset in synucleinopathies: a retrospective analysis. Lancet Neurol..

[82] Kurata T (2007). Enhanced accumulation of phosphorylated alpha-synuclein in double transgenic mice expressing mutant beta-amyloid precursor protein and presenilin-1. J. Neurosci. Res..

[83] Duka T, Duka V, Joyce JN, Sidhu A (2009). Alpha-Synuclein contributes to GSK-3betacatalyzed Tau phosphorylation in Parkinson’s disease models. FASEB J..

[84] Anderson JP (2006). Phosphorylation of Ser-129 is the dominant pathological modification of alpha-synuclein in familial and sporadic Lewy body disease. J. Biol. Chem..

[85] Swirski M (2014). Evaluating the relationship between amyloid-beta and alpha-synuclein phosphorylated at Ser129 in dementia with Lewy bodies and Parkinson’s disease. Alzheimers Res. Ther..

[86] Obi K (2008). Relationship of phosphorylated alphasynuclein and tau accumulation to Abeta deposition in the cerebral cortex of dementia with Lewy bodies. Exp. Neurol..

[87] Walker DG (2013). Changes in properties of serine 129 phosphorylated α-synuclein with progression of Lewy-type histopathology in human brains. Exp. Neurol..

[88] Galvin JE, Pollack J, Morris JC (2006). Clinical phenotype of Parkinson disease dementia. Neurology.

[89] Jellinger KA (2012). Neuropathology of sporadic Parkinson’s disease: evaluation and changes of concepts. Mov. Disord..

[90] Milber JM (2012). Lewy pathology is not the first signof degeneration in vulnerable neurons in Parkinson disease. Neurology.

[91] Kanazawa T (2012). Pale neurites, premature α-synuclein aggregates with centripetal extension from axon collaterals. Brain Pathol..

[92] Schulz-Schaeffer WJ (2012). Neurodegeneration in Parkinson disease: moving Lewy bodies out of focus. Neurology.

[93] Schulz-Schaeffer WJ (2015). Is cell death primary or secondary in the pathophysiology of idiopathic Parkinson’s disease?. Biomolecules.

[94] Bousset L (2013). Structural and functional characterization of two alpha-synuclein strains. Nat. Commun..

[95] Guo JL (2013). Distinct α-synuclein strains differentially promote tau inclusions in neurons. Cell.

[96] Chung CY, Koprich JB, Siddiqi H, Isacson O (2009). Dynamic changes in presynaptic and axonal transport proteins combined with striatal neuroinflammation precede dopaminergic neuronal loss in a rat model of AAV alpha-synucleinopathy. J. Neurosci..

[97] Ip CW (2017). AAV1/2 - induced overexpression of A53T-α-synuclein in the substantia nigra results in degeneration of the nigrostriatal system with Lewy-like pathology and motor impairment: a new mouse model for Parkinson’s disease. Acta Neuropathol. Commun..

[98] Gratwicke J (2013). The nucleus basalis of Meynert: a new target for deep brain stimulation in dementia?. Neurosci. Biobehav. Rev..

[99] Hale MW, Lowry CA (2013). Functional topography of midbrain and pontine serotonergic systems: implications for synaptic regulation of serotonergic circuits. Psychopharmacology.

[100] Zaja-Milatovic S (2005). Dendritic degeneration in neostriatal medium spiny neurons in Parkinson disease. Neurology.

[101] Stephens B (2005). Evidence of a breakdown of corticostriatal connections in Parkinson’s disease. Neuroscience.

[102] Goker-Alpan O (2012). The neurobiology of glucocerebrosidase-associated parkinsonism: a positron emission tomography study of dopamine synthesis and regional cerebral blood flow. Brain.

[103] Nalls MA (2013). A multicenter study of glucocerebrosidase mutations in dementia with Lewy bodies. JAMA Neurol..

[104] Bras J (2014). Genetic analysis implicates APOE, SNCA and suggest lysosomal dysfunction in the etiology of dementia with Lewy bodies. Hum. Mol. Gen..

[105] Schrag A, Siddiqui UF, Anastasiou Z, Weintraub Schott JM (2017). Clinical variables and biomarkers in prediction of cognitive impairment in patients with newly diagnosed Parkinson’s disease: a cohort study. Lancet Neurol..

